# The principles of good eye care research

**Published:** 2023-01-30

**Authors:** Clare Gilbert, GVS Murthy

**Affiliations:** Professor of International Eye Health: International Centre for Eye Health, London School of Hygiene & Tropical Medicine, London, UK.; Director: Indian Institute of Public Health, Hyderabad, India.

## Abstract

Eye health research is important for closing the gaps in our knowledge, but it should be ethical and based on sound scientific principles.

The purpose of research is to fill the gaps in our knowledge by providing evidence that we can trust and build on. This applies to all areas of research, whether astrophysics, education, housing, or health.

**Figure:**
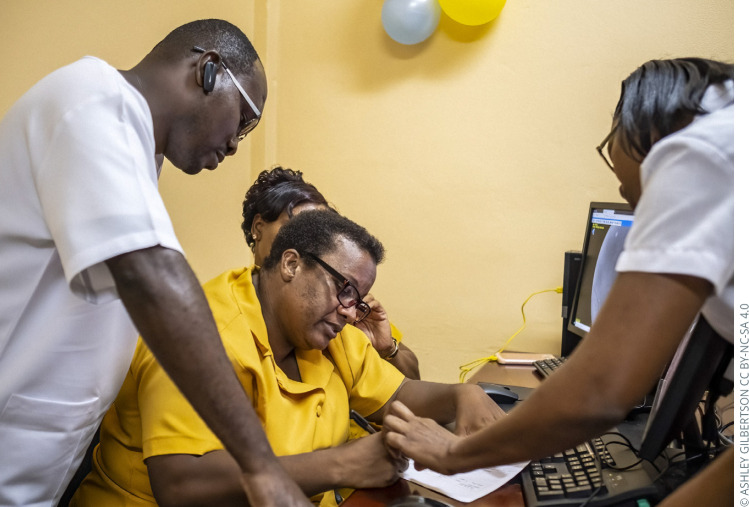
Involving several researchers helps to improve objectivity. st lucia

Well-conducted health research provides a solid basis for decision-making in clinical management, planning of health services, and deciding what further studies are needed. In the absence of research evidence, we tend to make decisions based on our own experiences and assumptions or based on what our more senior colleagues say. However, if we want to give our service users the best possible care, it is important to realise that our own experience – and that of our senior colleagues – may be limited, subjective, or even biased.

Making decisions on the basis of sound evidence, whenever possible, is far more effective and ethical.

Unfortunately, most eye health research is undertaken in high-income countries, and the findings may not apply to other settings for a number of reasons – e.g., ethnic differences in disease presentation and treatment. Undertaking local research, which addresses local needs and knowledge gaps, is essential.

## Research starts with a question

Regardless of the type of research, a critical first step is to form a research question, which may or may not be based on a hypothesis (defined as a supposition or proposed explanation, made on the basis of limited evidence, as a starting point for further investigation). The question should be clear, specific, and concise. Getting the question right is of critical importance, as the research objectives and methods, as well as the kind of participants to be recruited, depend on the research question.

## Gathering data

The data collected in studies can be quantitative, such as the number of people who attend for cataract surgery and their age and sex; or qualitative, such as asking people for their opinions or views on a particular topic.

Sometimes, quantitative and qualitative methods are both helpful. As an example, let's suppose that a high proportion of people identified in outreach with operable cataract do not attend for cataract surgery. We may assume that the costs of transport and surgery are the main problems. But, despite the offer of free transport and free surgery only half of these people come for surgery. The research question could be: **What are the characteristics of people with operable cataract identified in outreach who do not attend for surgery at the base hospital and why do they not attend?** The aim would be “to describe the characteristics of people with operable cataract identified during outreach who do not access cataract surgery compared with those who do, and to identify the reasons they give for not accessing surgery”. The first group of participants would need to be traced, as they did not attend for surgery.

This research question has two parts:

1. **Who are the people who do and do not access surgery?**
2. **Why do some people not access surgery?**

To answer part 1 of the research question above, a questionnaire would need to be designed to collect data on the age, sex, and circumstances of people attending and not attending for cataract surgery. Medical records could be used for patients who do undergo surgery, but routinely collected hospital data may not provide all the information needed. To find out more about who is not coming for surgery, a survey could be done to find out more about both groups of patients, e.g., their marital status, socioeconomic status, place of residence, level of education, the size of their household, the gender of the head of their household, etc. This is a **quantitative study**.

To answer part 2, the same questionnaire could include questions about possible reasons that patients did or did not attend for surgery. There could be a list of possible reasons (decided by the researchers) that participants who did not undergo surgery can respond to with either a ‘yes’ or ‘no’, or a list from which they can select one or more options. This approach allows the most important reasons to be determined, as the data collected are quantitative (i.e., the answers can be counted). The limitation of this approach is that the possible reasons participants can choose from will be limited by the researchers’ current understanding of the most likely or common reasons.

A way to find out more is to use **qualitative methods**, e.g., interviewing people who did not come for surgery and asking them open-ended questions. For example, it would be useful to know what they understand about their eye condition and what caused it, as these factors greatly influence how people behave. So a question could be: “Please can you explain what you think is wrong with your eyes?” followed by “What do you think caused your eye problem?” And then, “Please can you tell me why you did not attend the hospital for cataract surgery?” After a reason has been given, the next question can be: “Are there any other reasons?” The advantage of this type of data is that participants can also be asked how they made their decision about the surgery, whether anything or anyone influenced their decision and how, and what might help them to access cataract surgery. What the participants say in the interviews is then carefully analysed to identify the main reasons and solutions, which can inform further action to improve uptake.

It is important to note that quantitative and qualitative study designs require and use different research methods, which influence how many participants are required, how they are selected, and how data are collected and analysed. Quantitative and qualitative studies are not, therefore, interchangeable as they answer different research questions; the best way is to use both, as they complement each other. In this example, researchers could select a smaller group of patients who did not attend and have informal, qualitative discussions with them – either individually or in small groups (known as focus groups) – to discover possible reasons for non-attendance. These reasons can then be used in a quantitative survey, which is quick to administer and can be more easily analysed.

## Being objective

When conducting research of any kind, it is important to be objective, which means that you do not start the research with preconceived ideas about what the results might show – an open mind is essential. This means that the data collected, whether quantitative or qualitative, should not be influenced by the researchers at any stage in the research process, i.e., when the study is being designed and planned, or while data are being collected, analysed, and interpreted. These influences can lead to bias, which is defined as any systematic error in the design, conduct, or analysis of a study. For example, bias can occur if study participants are not appropriately selected, and care is not taken in how data are collected from them. There are several ways to avoid or reduce bias in health research, which include, but are not limited to, the following:

- Involve several researchers in the study from the outset and discuss every aspect. Ask experienced, independent researchers for their opinions.
- Carefully design all questionnaires and interview questions so that they are clear and unambiguous, and do not lead participants to respond in a particular way. These should be tested on a small number of participants first (known as pilot testing). The questionnaires or interview questions can be then modified, if necessary, before the main study takes place.
- Carefully select and rigorously train the fieldworkers who will collect the data, and monitor their performance during data collection. Poorly performing field workers may need to be retrained or replaced.
- Carefully select study participants to make sure they represent the group of people with the health condition or problem being investigated (important in quantitative studies) or are likely to reflect a range of perspectives (in qualitative research).
- Always take objective measurements whenever possible: e.g., take images of the retina that are later graded by experts or trained graders, rather than relying on clinical grading.
- Decide exactly how data will be analysed before data collection takes place, and keep to the analysis plan.
- Always report all the key findings of a study, even if they surprise or disappoint you.

“Do not start the research with preconceived ideas about what the results might show – an open mind is essential.”

## Ethics

As in all research involving people, the ethical implications need to be carefully considered from the start. For example:

- Ensure the study is of high scientific value and researchers have the skills to deliver all aspects of it.
- Take informed consent from all participants to ensure they fully understand the study, the procedures, and possible side effects/harm.
- Take particular care when obtaining consent from vulnerable groups, which include children, the very elderly, the very sick, those with a mental health condition, and individuals in institutions.
- Make participants aware that they are free to leave the study at any time without having to give a reason.
- Protect the anonymity of study participants by asking for their consent to record interviews (if interviewing) and using anonymous quotes.
- Maintain strict confidentiality of all data (text files, databases, images, etc.) by using password-protected computer storage with access restricted to the researchers only.
- Ensure that compensation for participants (if being considered) is not so large as to persuade them to take part against their better judgement, but is enough to cover out-of-pocket costs (e.g., travel).
- Obtain approval from the relevant ethics committee or institutional review boards.
- Always provide services for those with a clinical need for care (the principle of ‘no science without service’).

## Conclusions

Broad-ranging eye health research is required to provide evidence on which to base clinical decisions. Studies must be of a high scientific and ethical standard, and be conducted in a rigorous manner at every stage: from designing the study, through to collecting, analysing, and interpreting the data, and writing up the findings for dissemination. This applies to all studies, regardless of size.

